# Effects of Wheat Biscuits Enriched with Plant Proteins Incorporated into an Energy-Restricted Dietary Plan on Postprandial Metabolic Responses of Women with Overweight/Obesity

**DOI:** 10.3390/nu16081229

**Published:** 2024-04-20

**Authors:** Maria-Christina Kanata, Amalia E. Yanni, Chrysi Koliaki, Irene Pateras, Ioanna A. Anastasiou, Alexander Kokkinos, Vaios T. Karathanos

**Affiliations:** 1 Laboratory of Chemistry-Biochemistry-Physical Chemistry of Foods, Department of Nutrition and Dietetics, Harokopio University, 17671 Athens, Greece; mckanata@hua.gr (M.-C.K.); vkarath@hua.gr (V.T.K.); 2 First Department of Propaedeutic Internal Medicine, Medical School, National and Kapodistrian University of Athens, Laiko General Hospital, 15772 Athens, Greece; ckoliaki@yahoo.com (C.K.); anastasiouiwanna@gmail.com (I.A.A.); akokkinos@med.uoa.gr (A.K.); 3 ELBISCO S.A., Industrial and Commercial Food Company, 21st Km Marathonos Avenue, 19009 Pikermi, Greece; e_patera@elbisco.gr; 4 Department of Pharmacology, Medical School, National and Kapodistrian University of Athens, 15772 Athens, Greece

**Keywords:** obesity, weight loss, biscuits, plant proteins, L-arg, BCAAs, glycemic regulation, gut peptides, appetite

## Abstract

This study investigates the effect of daily consumption of wheat biscuits enriched with plant proteins in postprandial metabolic responses of women with overweight/obesity who follow an energy-restricted diet. Thirty apparently healthy women participated in a 12-week randomized controlled trial and were assigned either to a control (CB) or an intervention (PB) group. Participants consumed daily either a conventional (CB) or an isocaloric wheat biscuit enriched with plant proteins (PB) containing high amounts of amino acids with appetite-regulating properties, i.e., BCAAs and L-arg. At baseline and the end of the intervention, a mixed meal tolerance test was performed. The responses of glucose, insulin, ghrelin, GLP-1, and glicentin were evaluated over 180 min. After 12 weeks, both groups experienced significant decreases in body weight, fat mass, and waist circumference. In the PB group, a trend towards higher weight loss was observed, accompanied by lower carbohydrate, fat, and energy intakes (*p* < 0.05 compared to baseline and CB group), while decreases in fasting insulin and the HOMA-IR index were also observed (*p* < 0.05 compared to baseline). In both groups, similar postprandial glucose, ghrelin, and GLP-1 responses were detected, while iAUC for insulin was lower (*p* < 0.05). Interestingly, the iAUC of glicentin was greater in the PB group (*p* < 0.05 compared to baseline). Subjective appetite ratings were beneficially affected in both groups (*p* < 0.05). Consumption of wheat biscuits enriched in plant proteins contributed to greater weight loss, lower energy intake, and insulin resistance and had a positive impact on postprandial glicentin response, a peptide that can potentially predict long-term weight loss and decreased food intake.

## 1. Introduction

Obesity is defined as the situation in which a person accumulates excessive body fat, and it is considered a risk factor for numerous chronic diseases, including cardiovascular diseases (stroke, coronary heart disease), hypertension, dyslipidemia, type II diabetes mellitus (T2DM), some types of cancer and non-alcoholic fatty liver disease (NAFLD) [1]. The increase in body fat mass is linked with dysregulation of the homeostatic appetite system by causing alterations in the secretion of gut-derived peptides, including the orexigenic hormone ghrelin and the anorexigenic peptides glucagon-like peptide-1 (GLP-1), pancreatic polypeptide YY (PYY), and glicentin. Gut-derived hormones play a key role in energy and glucose homeostasis regulation and are determinants of food intake and thus contribute to the perpetuation of the vicious cycle of obesity [2].

The cornerstone regarding the prevention and treatment of obesity and its comorbidities is lifestyle therapy, among which adherence to a reduced-calorie diet is the most effective intervention [3]. A moderate weight loss of 5–10% in people with obesity leads to improvement in metabolic function, such as abdominal fat reduction and enhanced insulin sensitivity [4]. Diet quality is also important for achieving and maintaining a healthy weight in the long term. One of the most well known and established healthy eating patterns is the Mediterranean Diet (MD). The MD is based on the consumption of locally grown, minimally processed foods originating from plants (i.e., fruits, vegetables, whole grains, legumes, nuts, seeds, and herbs), and allows for a limited intake of animal-derived products, whereas its primary fat source is olive oil [1,5].

Legumes are an easily available, safe, and affordable plant protein source, with a low energy density, low glycemic index (GI), high dietary fiber (soluble and insoluble), and very low fat content [6,7]. Legume consumption has been linked with greater weight loss, reduced body mass index (BMI) and waist circumference (WC), increased satiety levels, attenuation of postprandial glycemia, and, thus, reduced risk of appearance of obesity and related co-morbidities [8,9,10].

Legumes are incorporated into various new plant-based product formulations, by replacing part of wheat flour, mainly due to their beneficial effects on human health [6]. Legume flours are richer in protein and fiber and contain less rapidly digested starch compared to refined wheat flour, the most popular flour type [11]. The enrichment of wheat-based snacks, e.g., biscuits, with legume flours contributes to the reduction of postprandial glycemia. In addition, the combination of legumes, seeds, and wheat increases the concentrations of specific amino acids with appetite-regulating properties, such as Branched Chain Amino Acids (BCAAs) and L-arginine (L-arg) [12].

BCAAs, and especially L-leucine (L-leu), contribute to the stimulation of insulin secretion, and thus regulation of glucose metabolism, by activation of the specific signaling systems present in hypothalamic neurons, activated protein kinase, mTOR/AMPK, and mammalian targets of rapamycin/AMP [13,14]. Additionally, L-leu intake has been linked with a reduction of food intake and weight loss by controlling the release of neuropeptide Y/Agouti-related peptide (NPY/AgRP) and proopiomelanocortin (POMC) [15,16,17]. Regarding L-arg, it is a precursor in the synthesis of nitric oxide, the endogenous vasodilator that increases blood flow and promotes insulin-mediated glucose uptake, leading to improvement of insulin secretion [18]. L-arg is recognized by specific amino acid sensing receptors that are present in the gut [19,20] and its administration has been related to increased postprandial levels of anorexigenic peptides (GLP-1 and PYY) in humans with normal weight or obesity [21].

In a previous study carried out by our research group in overweight/obese subjects who followed a restrictive dietary plan, it was shown that the consumption of a wheat biscuit enriched with plant proteins originating from legumes and seeds containing high amounts of BCAAs and L-arg leads to greater weight loss and significant improvements in certain metabolic parameters compared to a common isocaloric wheat biscuit [22]. Since the results of that study were very promising, a new protocol was designed, aiming to evaluate the postprandial metabolic responses of female subjects with overweight/obesity to a mixed meal tolerance test (MMTT) after a 12-week restrictive dietary intervention supplemented with the plant protein-enriched biscuit [12]. The present study assessed potential beneficial outcomes in glycemic, insulinemic, and gut hormone responses related to appetite regulation as a result of the dietary intervention conducted.

## 2. Materials and Methods

### 2.1. Study Subjects 

The target study population comprised women with overweight/obesity (BMI: 25–36 kg/m^2^) of reproductive age (18–50 years), without diabetes, and with normal exercise and drinking habits. The exclusion criteria were pregnancy, lactation, history of chronic medical illness, such as cardiovascular, renal, liver, untreated thyroid disease, and use of nutritional supplements that could interfere with the results. In addition, subjects with excessive exercise habits, a history of alcohol and/or drug use, and psychiatric diseases that could affect the adherence to the diet plan were also ineligible to participate in the study. 

Thirty premenopausal women, mean age 38 ± 8.6 years with body mass index BMI = 29.8 ± 3.53 kg/m^2^ (mean ± SD) were enrolled and successfully completed the dietary intervention. The subjects were recruited by posters, electronic advertisements, and personal communication. Before entering the study, the subjects were given written information about the study protocol in detail and then they gave voluntary informed written consent for participation. The study protocol was reviewed and approved by both the Institutional Review Board/Ethics Committee of Harokopio University of Athens and of Laiko General Hospital. The study protocol registration number is ClinicalTrials.gov: NCT05213559.

### 2.2. Study Design

**Figure 1:**
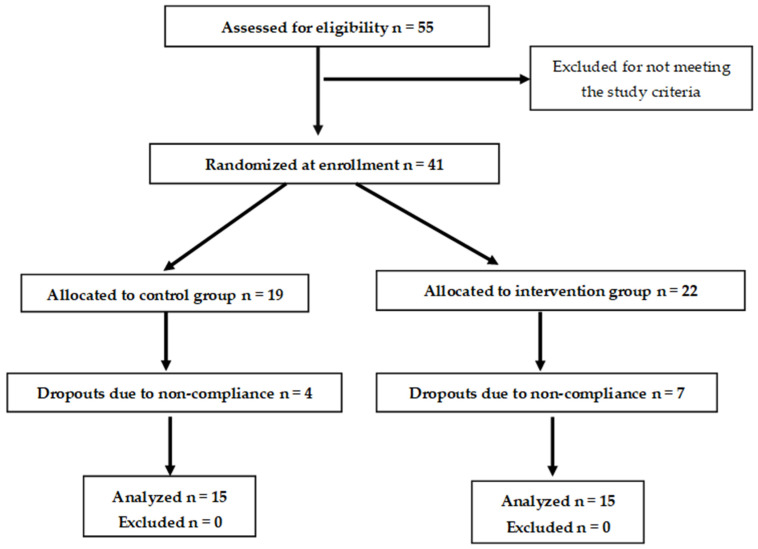
Flow chart of the study.

This study was conducted at the 1st Department of Propedeutic and Internal Medicine, Laiko General Hospital, Medical School, National and Kapodistrian University of Athens, in collaboration with the Laboratory of Chemistry–Biochemistry–Physical Chemistry of Foods, Department of Nutrition and Dietetics, Harokopio University of Athens, Greece. It was designed as a 12-week, single-blinded, randomized dietary intervention (Figure 1).

After a run-in period of 1-week duration, the participants were allocated, with the use of randomization software (www.randomizer.org, Research Randomizer, version 4.0, accessed on 15 October 2021), into either the control or the intervention group. Both groups were following a calorie-restricted diet, in which 80 g of biscuit enriched with plant proteins (PB) was incorporated, or a conventional isocaloric wheat biscuit (CB), which served as control. In both study groups, the volunteers received an initial personalized restrictive diet plan. Subjects came for follow-up sessions every 2 weeks, where they received individualized dietary advice from trained dieticians along with a diet plan. Participants were asked not to change their exercise habits during the study period. The individualized weekly diet plans of the participants were designed with a caloric deficit of 20% of their daily energy requirements. More specifically, the dietary plans were designed based on the MD with the following macronutrient distribution: 40–45% carbohydrates, 18–20% proteins, and 33–37% fat. The subjects were asked to follow their customized dietary plan for 12 consecutive weeks, along with the consumption of 5 biscuits daily (1 biscuit = 16 g), with 2–3 biscuits being consumed between breakfast and lunch and the rest as an afternoon snack. At each visit, participants were supplied with biscuits for 2 weeks.

Total daily intake was based on baseline anthropometric measurements (weight and height) and was calculated using the Schofield equation, multiplied by a PAL = 1.4, an indication of a relatively low physical activity level. The volunteers received their individualized dietary plans with the amount of each food product expressed in grams. For the food products that required cooking prior to their consumption, the quantity in grams was referring to the final cooked products (e.g., meat, fish, pasta, rice). The evaluation of the adherence to the dietary plan was conducted at each follow-up session by the measurement of weight, fat loss, reduction in circumferences (waist and hip), and by a 3-day weighed dietary record (two weekdays and one weekend day) for every study week. The volunteers who did not follow at least 75% of the prescribed diets for two successive visits were informed that they could not continue to participate in the study and were recorded as dropouts. During their baseline screening visit, participants were asked to complete questionnaires regarding, more specifically, demographic information, medical and diet history, energy intake, and expenditure. Energy intake was accessed by a semi-quantitative food frequency questionnaire (FFQ) in order to evaluate the participants’ dietary habits for the last three months. The energy expenditure was evaluated with the use of the International Physical Activity Questionnaire (IPAQ). The FFQ and IPAQ were also completed at the end of the 12-week study period.

On the first day of the 12-week study, period participants were asked to come to the laboratory in the morning between 8.00 and 9.00 am after an overnight fast. They underwent a detailed clinical examination and measurement of body weight and composition, which were also measured at their last visit at the end of the intervention. More specifically, the participants’ body weights were measured by an electronic scale and their body composition (fat and lean mass) was measured by the method of bioelectrical impedance analysis (Tanita BC-418, Tokyo, Japan). Height was measured by a stadiometer (Seca Mode 220, Hamburg, Germany), with the subjects being in a relaxed position, not wearing shoes and their arms hanging freely. The measurement of the waist circumference was conducted at the midpoint between the lower margin of the last palpable rib and the top of the iliac crest in a standing position at the end of gentle expiration. The hip circumference was measured at the widest portion of the buttocks. Both circumferences were measured in duplicate with a fiberglass tape.

At the beginning and the end of the intervention, a mixed meal tolerance test was performed. The participants were asked to refrain from strenuous exercise and alcohol consumption for at least 24 h prior to the test day and their food intake was individually standardized. An intravenous catheter was placed in the forearm vein, the subjects were allowed to rest for 10 min and the blood samples were drawn. Then, they were asked to consume a standardized meal, which consisted of 78 g of whole wheat bread, 80 g of cheese (16% fat), 72 g of turkey (2% fat), and 250 mL of orange juice. The macronutrient composition of the meal was 47% carbohydrates, 27% protein, and 26% fat, and yielded a total of 568 kcal. Because of the known impact of the menstrual cycle on appetite regulation, the baseline and the final measurements were conducted at the same phase of the menstrual cycle.

### 2.3. Blood Analyses

Fasting blood samples were collected both at the first and the last visit of the participants in EDTA-coated vacutainers, with the addition of dipeptidyl peptidase 4 (DPP4) inhibitor (10 μL/mL blood) and were immediately centrifuged at 4000 rpm for 10 min at 4 °C for plasma separation. In the case of serum, the blood samples were collected in plain tubes and were left at room temperature for 30 min, and were allowed to clot. After the separation of serum and plasma, the samples were stored at −80 °C until further analysis. 

Serum glucose concentrations were determined at 0, 30, 60, 90, 120, and 180 min postprandially. Basal biochemical measurements, including serum total cholesterol (TC), high-density lipoprotein cholesterol (HDL-c), triacylglycerols (TAG), alanine aminotransferase (ALT), aspartate aminotransferase (AST) and γ-glutamyl transferase (γ-GT) were also performed. All measurements were conducted with an automated biochemical analyzer using commercially available diagnostic kits (Medilyzer, Medicon Hellas S.A., Athens, Greece).

Insulin levels were measured in serum at 0, 30, 60, 90, 120, and 180 min postprandially by an Enzyme-linked Immunosorbent Assay (ELISA) Method (Human Insulin ELISA kit, Merck-Millipore, Burlington, MA, USA). The measurements of ghrelin were conducted with an ELISA method using a commercially available kit for human ghrelin (Human Ghrelin Total ELISA kit, Merck-Millipore, Burlington, MA, USA). For the determination of ghrelin, the plasma was pre-treated as it has been previously described [23]. Total GLP-1 and glicentin were also measured in plasma at 0, 30, 60, 90, 120, and 180 min by the sandwich ELISA Method (Human GLP-1 and Glicentin ELISA, Ansh Labs, Webster, TX, USA, respectively). Measurements were performed with Multiskan™ FC Microplate Photometer (Thermo Scientific, Waltham, MA, USA). 

### 2.4. Subjective Satiety Measurements

The evaluation of the subjective satiety measurements was performed with VAS (visual analog scale) booklets that were provided to the study participants. The questionnaires were filled before the mixed meal ingestion and after 30, 60, 90, 120, and 180 min postprandially, just before blood sample collection. The three questions that were included were “How hungry do you feel?”, “How full do you feel?” and “How great is your desire to eat?” A 10 cm line scale ranging from 0 (“not at all”) to 10 (“extremely”) expressing the most negative and the most positive rating, respectively, was used. The study participants were instructed to place a vertical mark on the line according to their feelings at this time and were not allowed to discuss their ratings with the other participants or refer to their own ratings from previous time points. The quantification was performed by measuring the distance (cm) from the left end of the line until the vertical mark in each of the three questions.

### 2.5. Test Biscuits

The PB was prepared by substitution of 30% of the white wheat flour with a mixture of flours originating from legumes and seeds and the CB was a conventional isocaloric wheat biscuit. The exact composition of the biscuits is presented in detail in a previous study [22]. It must be noted that the PB has a considerably higher protein content and individual amino acids originating from the plant flours compared to CB. The PB biscuit had twice the total amino acids content compared to CB and in terms of the amino acids that had the appetite-regulating properties, it was 75% richer in L-arg and 50% richer in BCAAs.

### 2.6. Calculations and Statistical Analysis

A power analysis was performed to calculate the sample size required for the intervention to have a statistically significant decrease of 1 mmol/L (18 mg/dL) in peak glucose concentration at the end of the dietary intervention. According to these assumptions, a total of 26 participants were required, 13 in each study group, with α = 0.05 and a power of 80%. To account for dropouts, fifteen participants per group were targeted for recruitment.

The incremental areas under the curve (iAUCs) for the postprandial glucose and insulin responses were calculated by the application of the trapezoidal rule, ignoring the area under the baseline [24]. The iAUC for ghrelin was expressed as a decrease from the preprandial values and was calculated using the iAUC, ignoring the area above the *x*-axis. The iAUC for the VAS scales expressed the differences from preprandial values and were calculated in a similar way. The HOMA-IR index was calculated using the equation:

HOMA-IR = [Fasting Glucose (mg/dL) × Fasting Insulin (μU/mL)]/405(1)

The comparison of the anthropometric and biochemical parameters at the beginning and the end of the intervention was conducted with a paired samples *t*-test. The differences between the two groups regarding the same parameters were evaluated by an independent samples *t*-test. The level of statistical significance was defined as *p* < 0.05. The SPSS 22.0 statistical software package (IBM, New York, NY, USA) was used for the analyses.

## 3. Results

### 3.1. Anthropometric and Biochemical Characteristics 

In total, 30 female subjects successfully completed the intervention, with 15 in the CB group and 15 in the PB group. In Table 1, the baseline anthropometric, biochemical, and clinical characteristics of the subjects are presented at the beginning and the end of the intervention. No statistically significant differences were observed between the participants of the two groups for all the examined parameters at the beginning of the study. After 12 weeks, a statistically significant reduction was observed in body weight, BMI, body fat and waist circumference in both groups (*p* < 0.05). There was a trend towards greater weight loss in the group that consumed the PB compared to CB, despite not being significant (percentage of weight loss: 5.31 vs. 4.24%).

In Table 2, the total energy and macronutrient intake, as evaluated by the FFQs, are presented. Regarding baseline values, no significant differences were observed in any variable between the two groups. Decreases were observed in both groups at the end of the 12-week intervention. However, greater reductions in total caloric, carbohydrate, and fat intake were observed in the PB group compared to the CB group (*p* < 0.05).

In addition, in the group that consumed the PB, a significant reduction in fasting insulin levels was observed at the end of the intervention (*p* < 0.05). A significant decrease was also recorded in HOΜA-IR in the same group (*p* < 0.05, Table 3).

### 3.2. Mixed Meal Tolerance Test

#### 3.2.1. Glucose and Hormone Responses

**Figure 2:**
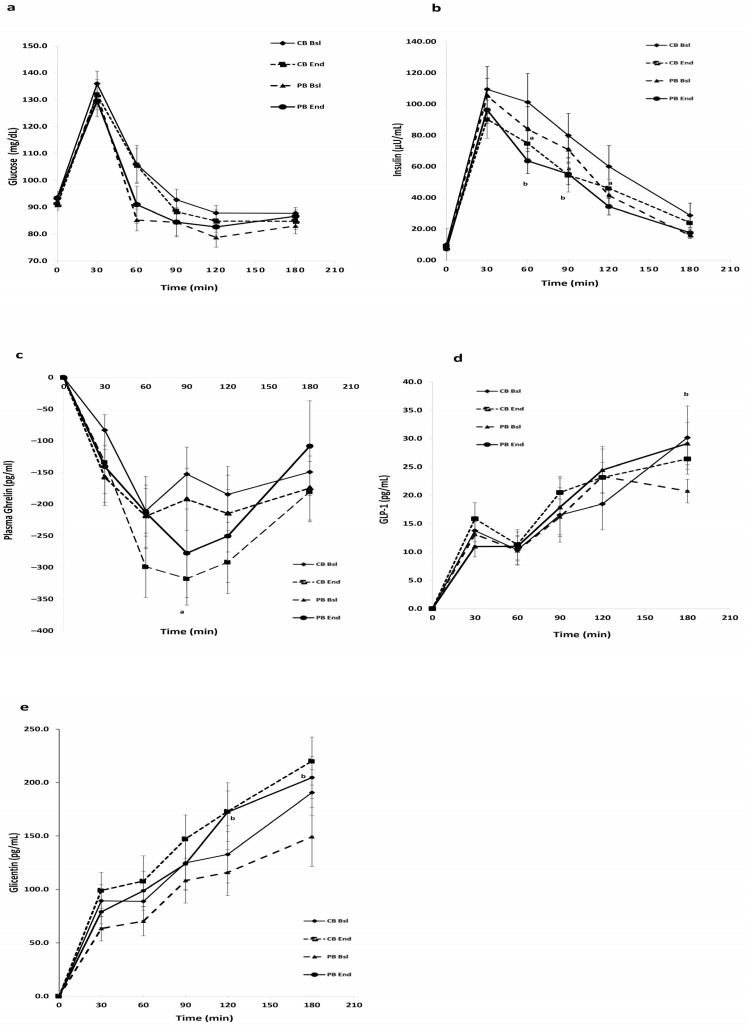
Postprandial glucose (**a**), insulin (**b**), ghrelin (**c**), GLP-1 (**d**), and glicentin (**e**) responses of the two groups of subjects during the mixed meal tolerance test, before and after the 12-week dietary intervention. Values are expressed as mean ± SΕΜ (n = 30). ^a^ *p* < 0.05 between CB Baseline and CB End, ^b^ *p* < 0.05 between PB Baseline and PB End.

The glycemic response for 180 min after the mixed meal consumption was similar at the beginning and the end of the 12-week dietary intervention in both CB and PB groups (Figure 2a). The incorporation of either the conventional biscuit or that enriched with plant proteins in the context of an energy-restricted dietary plan did not seem to affect the postprandial glycemia of the female subjects, as it was evaluated by the iAUC for 180 min after meal ingestion (Table 4). Regarding the insulin response, a significant reduction was found in the 180 min iAUC at the end of the intervention in both groups compared to baseline (*p* < 0.05 for CB and PB respectively, Figure 2b).

In addition, lower insulin levels were observed at 60, 90, and 120 min in the CB group and at 60 and 90 min in the PB group, respectively. The highest (peak) serum glucose and insulin concentrations were observed after 30 min of the mixed meal ingestion in both groups at baseline and the end of the intervention.

Total plasma ghrelin concentrations were reduced after the consumption of the mixed meal with the nadir values to be observed at 60 min at the beginning and 90 min postprandially after 12 weeks in both groups. No significant differences were observed at the end of the intervention for both groups in the iAUCs (Figure 2c, Table 4).

Regarding GLP-1, a significantly higher concentration was observed at 180 min in the PB group compared to baseline (*p* < 0.05, Figure 2d). No significant differences were found in any group or between groups in the iAUC values (Table 4). 

Glicentin postprandial response is presented in Figure 2e. There is a significant increase in the iAUC of glicentin after 180 min of meal ingestion in the PB group at the end of the intervention (*p* < 0.05, Table 4). In addition, a significant increase at 120 and 180 min was observed in the same group (*p* < 0.05).

#### 3.2.2. Subjective Appetite Ratings (VAS)

**Figure 3:**
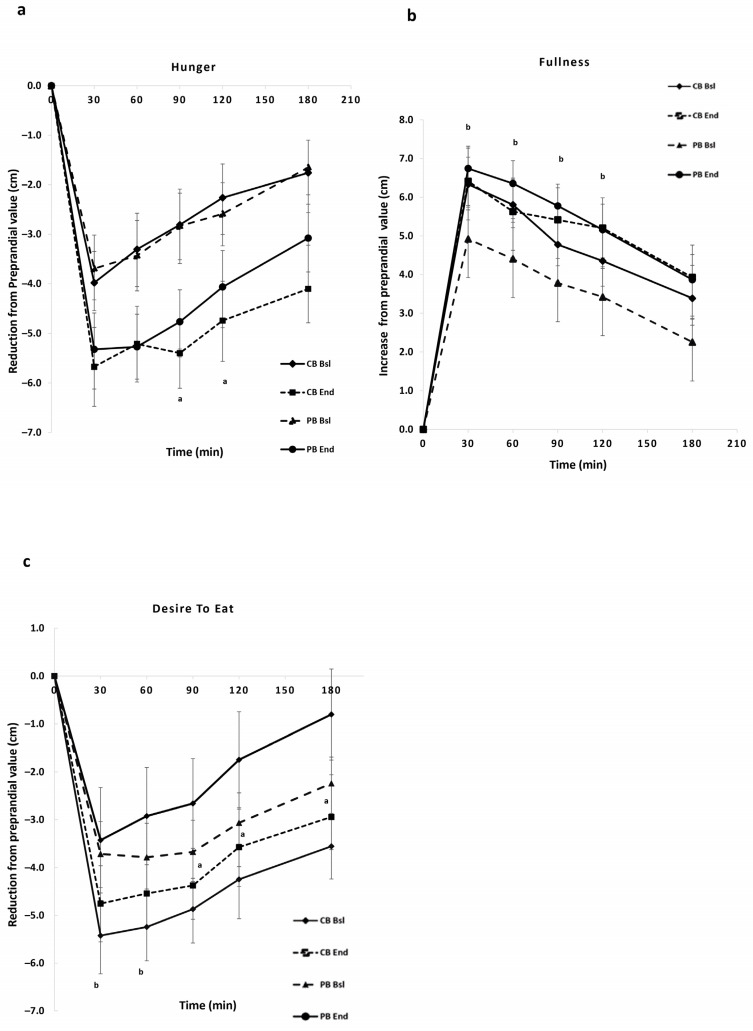
Subjective appetite ratings for hunger (**a**), fullness (**b**), and desire to eat (**c**) of the two groups of subjects during the mixed meal tolerance test, before and after the 12-week dietary intervention. Values are expressed as mean ± SEM (n = 30). ^a^ *p* < 0.05 between CB Baseline and CB End, ^b^ *p* < 0.05 between PB Baseline and PB End.

The subjective appetite ratings differences from the preprandial values for hunger, fullness, and desire to eat for 180 min after meal ingestion are shown in Figure 3. There was a significant reduction in iAUC for hunger at the end of the intervention in the CB group (*p* < 0.05, Table 5) and a trend towards a greater decrease in the PB group (*p* = 0.072) compared to baseline (Figure 3a). Certainly, in the CB group, a significant reduction was observed at 90 and 120 min and in the PB at 90 min (*p* < 0.05). In terms of fullness, a significant increase in iAUC for fullness values was recorded at the end in the PB group (*p* < 0.05, Table 5). In addition, greater fullness values were observed at 30, 60, 90, and 120 min (*p* < 0.05, compared to baseline, Figure 3b). The iAUC for desire to eat was significantly lower at 180 min in the CB group (*p* < 0.05, Table 5). In the CB group, there was also a greater decrease in postprandial values of desire to eat at 90, 120, and 180 min (*p* < 0.05), whereas, in the PB group, lower values were recorded at 30 and 60 min (*p* < 0.05, Figure 3c).

## 4. Discussion

The alarming increase in global obesity prevalence over the last decades, which has been closely associated with certain metabolic disorders, such as T2DM and dyslipidemia, is considered one of the major public health challenges [25]. There is an urgent need to find effective strategies for tackling obesity, both at the prevention and treatment levels. An energy-restricted and balanced dietary intervention such as the MD can lead to a significant reduction in body weight and beneficially impact the postprandial metabolic responses [26,27].

The increasing protein content of food products that have a traditionally low protein content can cause a greater increase in the postprandial levels of anorexigenic peptides released in the gut in response to food ingestion [21]. Increased legume consumption contributes to higher weight loss in humans with overweight/obesity, along with other beneficial impacts on health [28,29,30,31]. The exploitation of legume and seed flours for substitution of refined white wheat flour and the development of novel functional foods with lower GI and higher satiety potential can be an interesting new prospect in the food industry.

In the present study, both groups CB and PB were following a personalized restrictive dietary plan in combination with follow-up sessions with trained dieticians. First, the two study groups had followed a hypocaloric diet and had similar levels of physical activity, with the difference being the test food that was incorporated into their daily diet. The significant reduction in energy intake, in both groups, led to weight loss over the 12-week study duration, irrespectively of the intervention that each subject was allocated to. In addition, all subjects experienced beneficial changes in body fat mass and waist circumference, along with preservation of their lean mass, but there was no significant difference between groups. However, the PB had a trend towards a greater percentage of weight loss compared to the CB group (5.31 vs. 4.24%) and a higher waist circumference decrease (9.5 vs. 7.8 cm, respectively). The % weight loss difference between the two groups was similar to that of our previous study [22]. In our previous study, the percentages of weight loss were slightly higher. This can be attributed to two reasons. First, in that study, men, who tend to lose weight at a faster rate compared to women, also participated, and second, the number of participants in the present study was 30, less than half of those included in our previous study (70 subjects). From the power analysis calculation that was conducted in our previous work, it was found that 70 participants were required in order to detect a 2 kg difference in weight between the two groups. The present study was designed to investigate the differences in the postprandial metabolic responses after the intervention that required at least 26 participants. So, the lower number of participants may lead to a larger standard deviation between the subjects’ weight change, which is higher than the overall effect of PB consumption. However, the trend of the PB group for higher weight loss led to lower fasting insulin levels and HOMA-IR values after 12 weeks (*p* < 0.05), whereas in the CB group, no significant differences were observed. Weight loss of around 5% was considered to be sufficient for an improvement of *β*-cell function and insulin sensitivity [32]. Another reason could be related to the composition of the plant protein-enriched biscuit since it has been shown that consumption of biscuits enriched in L-arg, in the context of an energy-restricted diet, contributed to greater weight loss and exerted a beneficial impact on glucose metabolism and insulin sensitivity in subjects with obesity [18]. Furthermore, high dietary intake of L-arg originating from plant sources has been related to lower serum insulin levels, HOMA-IR values, and decreased risk of developing metabolic syndrome [33].

As has been already mentioned, a significantly greater reduction was recorded in the caloric intake in the PB group, which is in accordance with our previous research. The trend for higher weight loss in the PB group compared to the CB group can be explained by the higher reduction in caloric intake of the former. The higher reduction in caloric intake of the PB group can be explained by the consumption of biscuits enriched in plant proteins as a snack. First, the PB biscuit provided twice the amount of total proteins compared to the CB (11.6 g and 5.8 g/day, respectively), four times more L-arg (0.84 g and 0.19 g/day, respectively) and higher than twice the BCAAs (1.64 g and 0.78 g/day, respectively). Apart from the different biscuit composition, mainly in terms of protein quantity and contents of the specific amino acids, the two biscuits were isocaloric (360 kcal/day) and were consumed as morning and evening snacks (five biscuits in total) between the main meals by the volunteers of both groups. Moreover, all subjects received personalized advice from trained dieticians in combination with a specific customized weekly diet plan, which provided the same macronutrient distribution.

BCAAs and L-arg have been linked with enhanced appetite-regulating properties. In our previous study, an acute single-blinded randomized control crossover study, the two biscuits were consumed on two different occasions at a quantity yielding 50 g of available carbohydrates. The biscuit enriched in plant proteins originating from legumes and seeds with high amounts of L-arg and BCAAs was found to have a lower GI and increased postprandial GLP-1 and glicentin responses compared to the control wheat biscuit. In addition, significantly higher subjective feelings of fullness and lower hunger and desire to eat were recorded. Furthermore, its consumption elicited higher postprandial levels of plasma L-arg and BCAAs concentrations [12]. L-arg can be recognized by specific amino acid sensing receptors in the gut and contribute to the release of anorectic gut hormones such as GLP-1 [19,20]. In addition, L-arg has been related to a reduction in the rate of gastric emptying in human interventions [34]. The oral administration of L-arg in obese mice has been found to stimulate the secretion of GLP-1 and PYY, thus improving the glycemic response and reducing food intake [34,35]. In terms of human interventions, it has been found that oral ingestion of 3 g of L-arg before an ab libitum meal in human subjects led to a significant increase in postprandial GLP-1 and PYY levels [21]. Enrichment of bread with flour rich in L-arg has been shown to decrease GI and exert an insulinotropic action [36]. BCAAs and especially higher L-leu can suppress the release of the orexigenic neuropeptide Y/Agouti-related peptide (NPY/AgRP) and proopiomelanocortin (POMC) and thus has been linked with enhanced weight loss and decrease in food intake [15,16,17]. In an acute postprandial study, in which healthy women participated, the consumption of snacks (cereal bars) enriched with 2 g of L-leu positively impacted subjective appetite sensations, leading to increased fullness, decreased hunger, and a desire to eat for healthy women participants [15].

In the present study, a MMTT was conducted at baseline and the end of the dietary intervention with the aim of evaluating the potential benefits of the PB in the postprandial metabolic response in terms of glycemia, insulinemia, and appetite regulation. The MMTT replicates more closely the digestion and absorption of complex foods consumed daily and mimics the physiological metabolic response. It can be effective in the detection of disorders in glucose homeostasis, the secretion of insulin, and stimulation of the incretin axis. The MMTT provides all macronutrients, and the stimulation of insulin can also be caused by certain amino acids and fatty acids apart from glucose [37,38].

The postprandial glycemia seemed unaffected both by the dietary intervention, as well as the type of biscuit that was consumed by the volunteers. However, it must be noted that the glycemic response was in the normal range at the baseline of the intervention. Participants were normoglycemic, non-diabetic, and apparently healthy with mild obesity. In both groups, the percentage of body weight they lost was not very high (max 5.3%) to induce a significant change in glucose response. In another study, it has been found that 10% of weight loss after a non-energy-restricted diet significantly reduced postprandial glucose levels. So, if the duration of the intervention was more than 12 weeks and subjects could have lost more weight, then significant changes might have been observed [27]. On the other hand, the postprandial insulinemic response was significantly lower in both groups, showing that weight loss favorably affected insulin levels, irrespective of the type of biscuit consumed. With postprandial glucose being unaffected by the intervention, it can be assumed that less insulin is required for glucose homeostasis after weight loss of approximately 5%. Postprandial ghrelin levels after ingestion of a balanced meal do not seem to be affected either by weight loss in this amount or by the administered biscuit composition.

GLP-1 is secreted by the L-cells present in the small intestine and colon in response to meal ingestion. The L-cells express specific nutrient-sensing receptors, such as G-protein-coupled receptors and molecular transporters that recognize nutrients in the luminal content. GLP-1 is an anorectic peptide that belongs to incretins, which stimulates the release of insulin and delays the rate of gastric emptying postprandially [39]. As shown in Figure 2d, the postprandial GLP-1 response of both groups can be further split into two phases, observed at the beginning and the end of the intervention. First, the typical early postprandial phase with a peak value at 30 min and a second later prolonged postprandial phase at which GLP-1 levels start to rise again at 90 min with the most probable second peak value appearing at 180 min. That second sustained rise of plasma GLP-1 levels can be mainly attributed to the large meal size and the presence of still unabsorbed nutrients and other metabolites (e.g., secondary bile acids, short-chain fatty acids, and microbial metabolites) at the distal ileum and colon, where there is a dense distribution of L-cells that secrete GLP-1 [40]. It has been already reported that proglucagon-derived peptides (PGDP) postprandial concentrations are influenced by the energy content of the meal and that a relatively high-calorie meal (~600 kcal) induces a prolonged response of GLP-1 and glicentin that may persist up to the 180 min completion and can last up to 300 min [41]. That second GLP-1 response does not require glucose absorption by sodium/glucose cotransporter-1 (SGLT-1) and can also affect the rate of digestion as a result of glucose appearance in the circulation at the subsequent meal [9]. The postprandial GLP-1, as measured by iAUC, did not change at the end of the intervention in both groups. Additionally, no significant difference was found between groups at the end of the intervention. However, at 180 min, the second peak, a significant difference was found in the PB group (*p* < 0.05), indicating that probably the GLP-1 response from 180 min until it returned to baseline levels could be significantly higher than the baseline. This higher response may be important because it can affect the subsequent meal energy intake.

The determination of GLP-1 concentration is challenging and is not enough alone for the evaluation of the postprandial metabolic response. The main reason is that its circulating concentrations are generally low and the postprandial increase after food intake (principal stimulus) is very modest, thus making it more difficult to detect all the potential changes in its postprandial levels [42]. Regarding plasma postprandial glicentin levels, they were significantly higher in the PB group, which is an important finding since recently, glicentin levels have been linked with weight management. Glicentin is a 69-amino acid peptide, which is cleaved from proglucagon and contains the entire sequence of oxyntomodulin. It is synthesized in the distal small bowel and proximal colon, and it is co-secreted by the L-cells (as GLP-1). Glicentin plays a significant role in glucose homeostasis by having an insulinotropic action and exerting an incretin-like effect. Additionally, it can contribute to appetite inhibition and reduction of glucagon secretion [43,44]. Its secretion is stimulated by food intake and, more specifically, by the elevated levels of glucose, lipids, and amino acids in the duodenum postprandially. In terms of amino acids, the intraluminal administration of a mixture of amino acids, including L-leu, isoleucine, and valine significantly enhanced postprandial glicentin levels. Similarly, oral administration of L-arg also led to higher postprandial glicentin response, suggesting that they are capable of simulating glicentin secretion from the L-cells in the small intestine [43]. In obesity, glicentin secretion is altered and it has been found that subjects with obesity have lower fasting glicentin levels compared to lean individuals. Changes in plasma glicentin levels can also mirror changes in GLP-1 and can be a superior marker for monitoring L-cell secretory activity affecting overall metabolic health in general. The main reason is that GLP-1 is rapidly eliminated from circulation, whereas glicentin has a longer half-life and reaches significantly higher postprandial levels [45]. Glicentin has been found to be one of the best predictors of a decrease in energy intake, the preference for energy-dense foods, and weight loss in patients who have undergone bariatric surgery [46,47]. It was found that the increase in % change of AUC of glicentin in the first three months was associated with weight loss after 12 months in bariatric surgery patients [46]. The accurate measurement of plasma glicentin has been very complicated, since there were no reliable methods, due to its cross-reactivity with other peptides. However, the recent advances in its detection can make glicentin a valuable tool as a non-invasive biomarker for metabolic disorders, such as obesity and T2DM [44].

The current study is a human study, and it has some limitations, with the most important being the subjects’ compliance with the dietary intervention. The extent of adherence to the dietary plan plays a significant role in the extent of weight loss achieved, regardless of the type of dietary intervention. So, it would be very useful in future studies to use validated measures of adherence for dietary interventions [48,49]. An interesting future perspective would be the execution of a similar dietary intervention with a longer duration that would lead to greater weight loss and, as a result, the magnitude of change of certain biochemical and metabolic parameters would be more prominent. In addition, further research can be conducted to assess whether the beneficial effects of the intervention (weight loss and metabolic improvements) are sustained in the long term and if it can potentially contribute to further weight loss. So, it would be an interesting perspective for further research in the future to include long-term follow-up for the evaluation of the sustainability of the positive effects that resulted from the interventions. So, another study can confirm and highlight the results of the current one.

In conclusion, the results of the present study indicate that the consumption of a plant-based biscuit that contains high amounts of amino acids with appetite-regulating properties in the context of a hypocaloric diet plan increases the participants’ adherence and contributes to lower energy intake and higher weight loss. Additionally, the decreased fasting insulin and HOMA-IR values in the group that consumed the enriched biscuit accounts for an increase in insulin sensitivity. An important new finding of this study is that the dietary intervention with the supplementation of the plant-based protein-enriched biscuit caused significantly higher circulating levels of glicentin during a MMTT, a peptide that can potentially predict the long-term weight loss as well as the reduction in food intake.

## Figures and Tables

**Table 1:** Anthropometric and biochemical characteristics of the subjects at baseline and after 12 weeks.

Variable	CB Baseline	CB End	*p*-Value *	PB Baseline	PB End	*p*-Value *
Age (years)	38.3 ± 8.0			37.8± 9.4		
Weight (kg)	80.4 ± 2.5	77.0 ± 2.8	**0.001**	80.2 ± 3.2	75.9 ± 3.2	**<0.001**
Body fat mass (kg)	31.4 ±1.6	27.9 ± 1.8	**0.001**	29.6 ± 2.9	25.8 ± 2.7	**<0.001**
Body fat (%)	38.7± 1.0	35.8 ± 1.2	**0.005**	37.7 ± 1.1	35.2 ± 1.3	**0.015**
BMI (kg/m^2^)	30.5 ± 0.9	29.0 ± 1.0	**0.006**	29.4 ± 1.0	27.9 ± 1.0	**<0.001**
WC (cm)	93.6 ± 1.9	85.8 ± 2.2	**<0.001**	89.5 ± 2.9	80.0 ± 2.5	**<0.001**
Lean mass (kg)	49.0 ± 1.2	49.1 ± 1.3	0.902	50.5 ± 2.2	50.8 ± 2.3	0.452
Glucose (mg/dL)	92.9 ± 1.5	90.5 ± 2.0	0.146	91.4 ± 2.8	92.6 ± 2.5	0.453
Cholesterol (mg/dL)	183.5 ± 10.9	179.7 ± 7.3	0.565	179.3 ± 7.2	176.9 ± 6.6	0.709
HDL-c (mg/dL)	49.9 ± 2.6	49.1 ± 2.4	0.331	54.3 ± 3.1	55.0 ± 2.9	0.367
LDL-c (mg/dL)	114.4 ± 9.8	114.7 ± 6.7	0.968	111.20 ± 6.5	108.5 ± 7.1	0.662
Triacylglycerols (mg/dL)	83.7 ± 9.9	80.3 ± 9.5	0.654	67.7 ± 7.1	68.4 ± 6.4	0.871
AST (U/L)	16.7 ± 1.1	15.8 ± 1.1	0.380	15.8 ± 1.2	14.9 ± 0.7	0.337
ALT (U/L)	17.0 ± 1.8	16.3 ± 1.6	0.694	15.8 ± 1.2	14.9 ± 0.7	0.596
γ-GT (U/L)	18.1 ± 1.3	18.3 ± 1.2	0.902	17.2 ± 0.7	16.2 ± 0.9	0.149

Values are expressed as mean ± SD. * refers to *p*-value < 0.05 in each group compared to baseline. *p*-value was calculated via paired samples *t*-test. BMI; body mass index, WC; waist circumference, SBP; systolic blood pressure, DBP; diastolic blood pressure, HDL-c; high-density lipoprotein cholesterol, LDL-c; low-density lipoprotein cholesterol, AST; aspartate aminotransferase, ALT; alanine aminotransferase, γ-GT; γ-glutamyltransferase.

**Table 2:** Energy and nutrient intake of the subjects of both groups at the beginning and after the 12-week dietary intervention.

Variable	CB Baseline	PB Baseline	*p*-Value *	CB End	PB End	*p*-Value *
Energy (Kcal)	2356.0 ± 396.3	2372.5 ± 416.8	0.912	1965.0 ± 153.0	1703.2 ± 39.5	**0.015**
Protein (g)	97.2 ± 26.2	97.8 ± 4.5	0.940	79.8 ± 22.3	81.6 ± 2.2	0.772
Carbohydrate (g)	247.4 ± 41.4	227.3 ± 61.7	0.306	232.8 ± 42.3	191.1 ± 19.6	**0.002**
Fat (g)	109.5 ± 26.1	117.3 ± 26.3	0.421	81.4 ± 16.9	69.8 ± 11.2	**0.036**

Values are expressed as mean ± SD. * refers to *p*-value < 0.05 between groups. *p*-value was calculated via independent samples *t*-test.

**Table 3:** Fasting Insulin levels and HOMA-IR index of both groups at the beginning and the end of the 12-week dietary intervention.

	CB Baseline	CB End	*p*-Value	PB Baseline	PB End	*p*-Value *	*p*-Value **
Insulin (μU/mL)	10.12 ± 1.60	8.97 ± 1.30	0.264	10.10 ± 1.56	7.30 ± 1.06	**0.020**	0.498
HOMA-IR	2.38 ± 0.49	2.08 ± 0.53	0.263	2.38 ± 0.43	1.71 ± 0.28	**0.018**	0.556

Values are expressed as mean ± SD. * refers to *p*-value < 0.05, *p*-value was calculated via paired samples *t*-test. ** refers to *p*-value < 0.05, *p*-value was calculated via independent samples *t*-test.

**Table 4:** iAUC of glucose, insulin, ghrelin, GLP-1, and glicentin responses over 180 min, before and after the 12-week dietary intervention in both groups.

iAUC-180 min	CB Baseline	CB End	*p*-Value *	PB Baseline	PB End	*p*-Value *	*p*-Value **
Glucose (mg·min·dL^−1^)	1837.06 ± 267.33	1728.45 ± 325.65	0.683	1283.42 ± 502.07	1248.00 ± 1004.00	0.900	0.264
Insulin (μU·min·mL^−1^)	10,629.00 ± 1770.76	7947.28 ± 1103.96	**0.004**	8786.55 ± 781.65	7178.30 ± 580.00	**0.009**	0.543
Ghrelin (pg·min·mL^−1^)	−25,106.36 ± 5138.34	−37,926.42 ± 7551.38	0.057	−35,666.4 7± 8473.14	−35,575.36 ± 6643.95	0.981	0.820
GLP-1 (pg·min·mL^−1^)	2976.00 ± 534.05	3262.00 ± 336.10	0.394	2878.00 ± 379.00	3185.86 ± 461.34	0.557	0.895
Glicentin (pg·min·mL^−1^)	20,873.72 ± 3113.35	25,034.95 ± 2734.00	0.138	17,809.83 ± 2634.46	24,086.62 ± 2966.63	**0.040**	0.484

Values are expressed as mean ± SEM. * refers to *p*-value < 0.05, *p*-value was calculated via paired samples *t*-test. ** refers to *p*-value < 0.05, *p*-value was calculated via independent samples *t*-test. iAUC; incremental area under the curve, GLP-1; glucagon-like peptide-1.

**Table 5:** iAUC of subjective appetite ratings responses (hunger, fullness, desire to eat) before and after the 12-week dietary intervention in both groups.

iAUC (cmx180 min)	CB Baseline	CB End	*p*-Value *	PB Baseline	PB End	*p*-Value *
Hunger	−561.27 ± 86.62	−828.06 ± 120.85	**0.036**	−545.66 ± 97	−750.20 ± 112.61	0.072
Fullness	806.62 ± 109.01	873.94 ± 128.00	0.504	680.37 ± 97	911.87 ± 93.54	**0.019**
Desire to eat	−587.52 ± 87.55	−891.36 ± 134.22	**0.021**	−598.28 ± 102.44	−736.34 ± 115.78	0.226

Values are expressed as mean ± SEM. * refers to *p*-value < 0.05. *p*-value was calculated via paired samples *t*-test.

## Data Availability

The raw data supporting the conclusions of this article will be made available by the authors on request.
